# Online teaching of inflammatory skin pathology by a French-speaking International University Network

**DOI:** 10.1186/1746-1596-9-S1-S5

**Published:** 2014-12-19

**Authors:** Emilie Perron, Maxime Battistella, Béatrice Vergier, Maryse Fiche, Philippe Bertheau, Bernard Têtu, the working group of the french College of Pathologists (CoPath/CUFP)

**Affiliations:** 1 Pathology department, Centre Hospitalier Universitaire (CHU) de Québec, and Faculty of Medicine, Université Laval, Québec, Canada; 2 Université Paris Diderot, Sorbonne Paris Cité, Laboratoire de Pathologie, UMR-S 1165, and AP-HP, Hôpital Saint-Louis, Department of Pathology, Paris, F-75010, France; 3 Service de Pathologie, Centre Hospitalier Universitaire (CHU) de Bordeaux et Université de Bordeaux, France; 4 Institut Universitaire de Pathologie, CHUV Lausanne, Switzerland; 5 Universities of Aix-Marseille, Angers, Besançon, Bordeaux, Caen, Grenoble, Montpellier, Nancy, Nantes, Nice, Paris Descartes, Paris Diderot, Paris Sud, Paris Est Créteil, Paris Pierre et Marie Curie, Paris 13, Poitiers, Rouen, Toulouse and Versailles St Quentin

## Abstract

**Introduction**

Developments in technology, web-based teaching and whole slide imaging have broadened the teaching horizon in anatomic pathology. Creating online learning material including many types of media such as radiologic images, whole slides, videos, clinical and macroscopic photographs, is now accessible to most universities. Unfortunately, a major limiting factor to maintain and update the learning material is the amount of resources needed. In this perspective, a French-national university network was initiated in 2011 to build joint online teaching modules consisting of clinical cases and tests. The network has since expanded internationally to Québec, Switzerland and Ivory Coast.

**Method**

One of the first steps of the project was to build a learning module on inflammatory skin pathology for interns and residents in pathology and dermatology. A pathology resident from Québec spent 6 weeks in France and Switzerland to develop the contents and build the module on an e-learning Moodle platform under the supervision of two dermatopathologists. The learning module contains text, interactive clinical cases, tests with feedback, virtual slides, images and clinical photographs. For that module, the virtual slides are decentralized in 2 universities (Bordeaux and Paris 7). Each university is responsible of its own slide scanning, image storage and online display with virtual slide viewers.

**Results**

The module on inflammatory skin pathology includes more than 50 web pages with French original content, tests and clinical cases, links to over 45 virtual images and more than 50 microscopic and clinical photographs. The whole learning module is being revised by four dermatopathologists and two senior pathologists. It will be accessible to interns and residents in the spring of 2014. The experience and knowledge gained from that work will be transferred to the next international resident whose work will be aimed at creating lung and breast pathology learning modules.

**Conclusion**

The challenges of sustaining a project of this scope are numerous. The technical aspect of whole-slide imaging and storage needs to be developed by each university or group. The content needs to be regularly updated and its accuracy reviewed by experts in each individual domain. The learning modules also need to be promoted within the academic community to ensure maximal benefit for trainees. A collateral benefit of the project was the establishment of international partnerships between French-speaking universities and pathologists with the common goal of promoting pathology education through the use of multi-media technology including whole slide imaging.

## Introduction

The field of anatomic pathology, a medical speciality dedicated to the study and the diagnosis of diseases based on the gross, microscopic, chemical, immunologic and molecular examination of organs, tissues, and whole bodies (autopsy) has been using computers for teaching for almost thirty years [1]. Developments in technology, web-based teaching and whole slide imaging have broadened the teaching horizon in anatomic pathology. Whole-slide imaging (WSI) is the digitization of entire glass slides at the same optical resolutions as light microscopy. WSI allows more interactivity, flexibility and easier access to interesting and educational cases for residents and pathologists [2]. The physical limitations of glass slide based study sets, such as deterioration of stain quality and breakage, can be circumvented by a web-based teaching module utilizing whole-slide imaging. Creating online learning material including radiologic images, videos, clinical and macroscopic photographs and whole slide images is now accessible to most universities. The structure of the learning process can be more interactive with clinical cases, direct links, tests and feedback. Similar educational methods have been used successfully in other domains of medical and health sciences [3-5].

Unfortunately, a major limiting factor to maintain and update the learning material is the amount of resources needed. In this perspective, a French national university network was initiated in 2011 to build joint online teaching pathology modules with clinical cases and tests [6]. The network has since expanded internationally to Québec, Switzerland and Ivory Coast. This report briefly describes the major aims of the project using a module on inflammatory skin pathology as an example.

## Method

The French national university network includes teachers from 19 universities across France. This network was used as a starting point for the international project. The national e-learning platform is hosted by PRES Sorbonne Paris Cité http://moodle.sorbonne-paris-cite.fr and is based on the free software *modular object-oriented dynamic learning environment *(Moodle). One of the first steps of the international project was to build an e-learning module intended for interns and residents in pathology. The first international fellowship was attributed to a pathology resident (EP) from Québec who spent 6 weeks in France and Switzerland to develop the contents and build the module with financial support obtained from CFQCU http://www.cfqcu.org/. This first module, created under the supervision of two dermatopathologists (BV, MB), focuses on inflammatory skin pathology. The broad range of pathologies, the direct clinical correlation and the interest of the resident for dermatopathology were key factors in choosing this topic.

To create the WSI, representative cases were selected and the de-identified glass slides were scanned at a 40× magnification using a Nanozoomer 2.0 RS or HT (Hamamatsu Photonics, *Shizuoka Prefecture, Japan*) and saved on a local dedicated pathology server. Each university was responsible for its own slide scanning, image storage and online display with virtual slide viewers (currently, mScope, Aurora Interactive Ltd., *Montreal, Canada*). For the inflammatory skin pathology module, the virtual slides are currently decentralized in two universities (Bordeaux and Paris 7). The clinicians and the medical imaging department provided the clinical images related to each case. Identifying features were removed with Paint software (Microsoft, Redmond WA). Signed consent was obtained from all patients.

Clinical cases along with explanations on each pathological lesion were built from prototypic and interesting cases seen by Drs Vergier and Battistella in their practice. The information provided was obtained from various textbooks [7-10].

## Results

**Figure 1:**
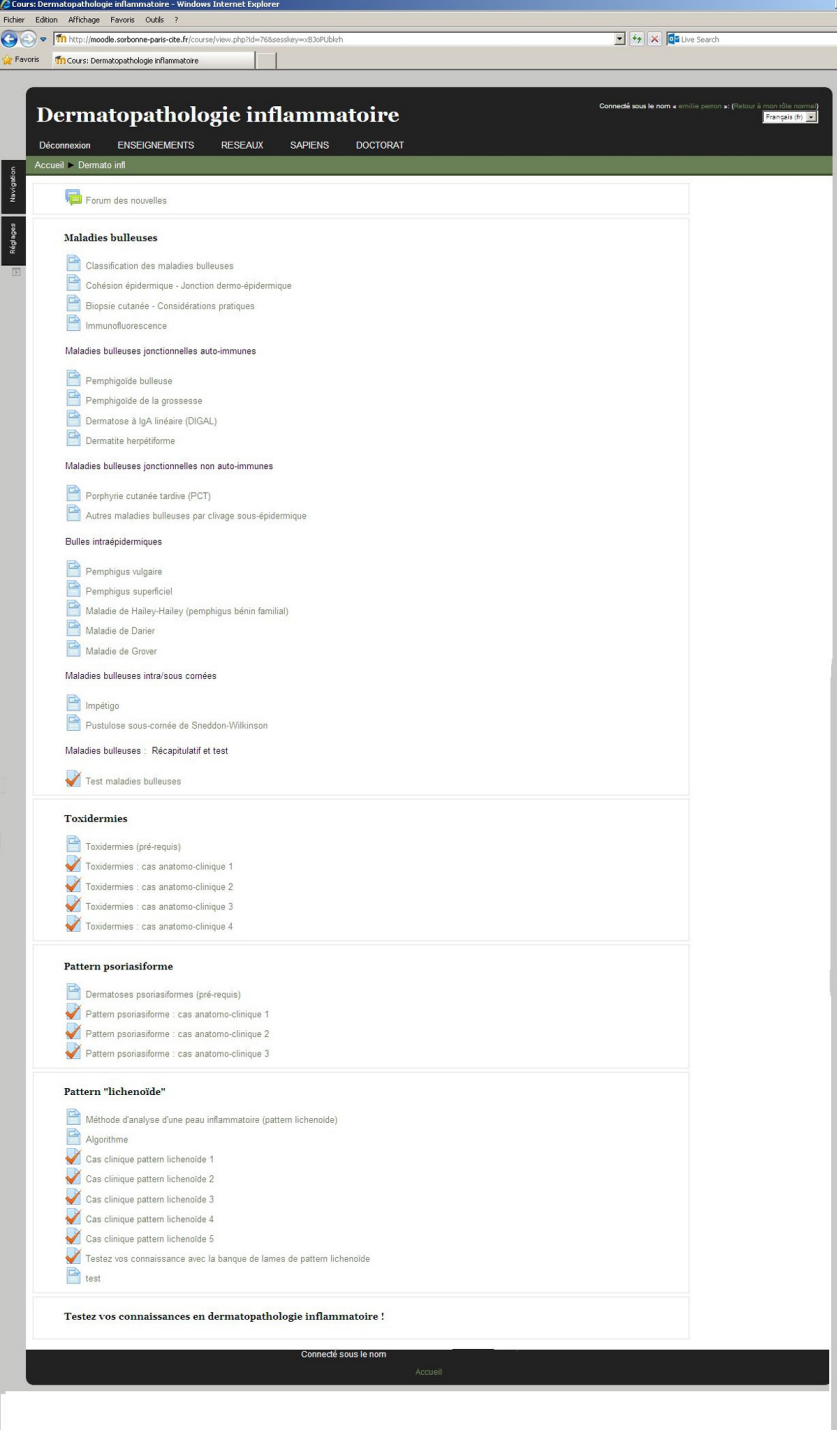
**The learning module content**.

**Figure 2:**
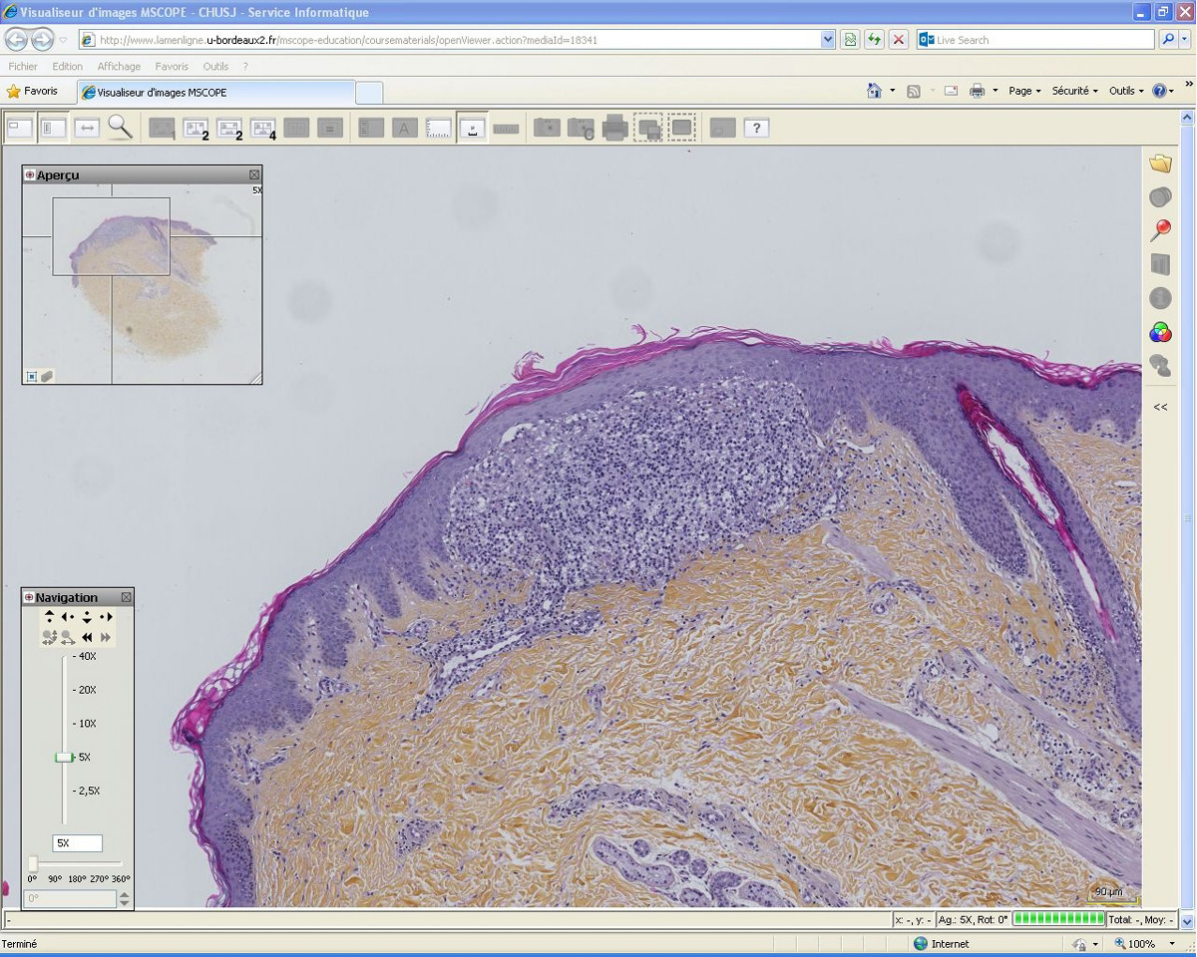
**WSI example**.

**Figure 3:**
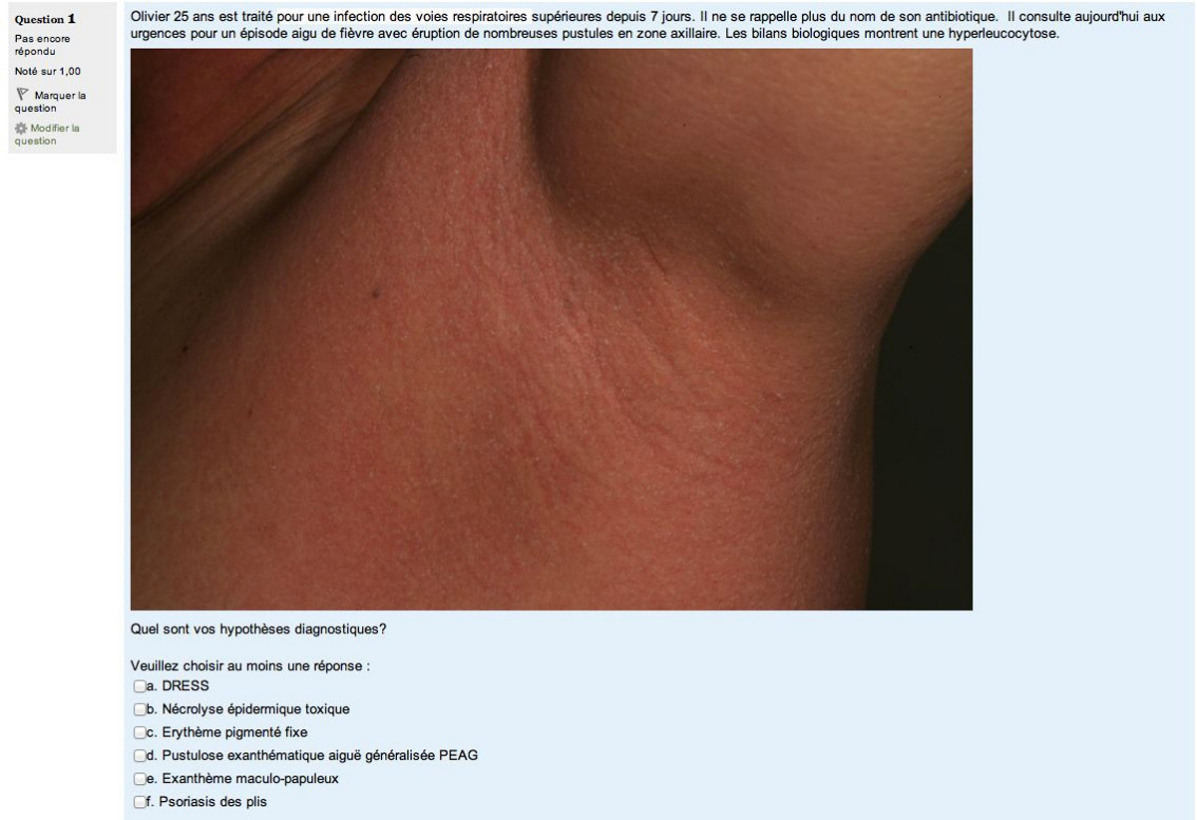
**Example of a section based on a case-study methodology**.

The learning module contains text, interactive clinical cases, tests with feedback, whole slides images, images and clinical photographs. The learning module is divided into 5 sections: bullous diseases, cutaneous drug reactions, psoriasiform reaction patterns, lichenoid reaction patterns and a general inflammatory skin pathology test (Figure 1). The first section, bullous diseases, is organised in a classic fashion. An introduction is given on the classification of bullous disease, epidermal cohesion, skin biopsy and immunofluorescence. Then, each of the characteristic disease is discussed using the following structure: epidemiology, pathogenesis, clinical features, microscopy, immunofluorescence, differential diagnosis and treatment. The links to the different pathologies and WSI (Figure 2) introduce the functionalities of the platform. Subsequently, a test feature is used to consolidate the notions learned by answering questions and identifying features on WSI. The cutaneous drug and psoriasiform reaction patterns sections are based on a case-study methodology. They provide standard descriptions of the pathologies followed by typical case studies with patient's history, relevant findings on examination, clinical photographs and interactive questions with WSI interpretation (Figure 3). The section on lichenoid reaction patterns uses a step by step analysis of the WSIs. The WSI is the first information provided with questions on its analysis and feedback. Then a differential diagnosis is requested and clinical information with photographs are given until a final diagnosis is reached. The pathology is then discussed using the structure previously described.

The module on inflammatory skin pathology includes more than 50 web pages with French original content, tests and clinical cases, links to over 45 WSI and more than 50 micro and clinical photographs. Three dermatopathologists and two senior pathologists are currently revising the entire learning module. It will be accessible to interns and residents in the spring of 2014. A survey of first-time users and subsequent focus groups with dermatology and pathology residents will be used to improve this learning tool. As demonstrated by Sun *et al*. [11], course quality is strongly associated with learners satisfaction. The quality of the material (WS, photography, etc.) and information provided has to be validated and adequate. Updates and additional material will be submitted through an editorial committee. The experience and knowledge gained from this work will be transferred to the next international fellow whose work will be aimed at creating lung and breast pathology learning modules.

## Conclusion

The challenges of sustaining a project of this scope are numerous. First, the technical aspect of whole-slide imaging and storage needs to be developed by each university or group. The creation of a committee of specialists to evaluate the course and feedback from users will be of utmost importance to orient the evolution of this project. The flexibility of this type of platform allows the e-learning content to be regularly updated, improved and maintained.

The accessibility and ease of use of the platform and whole-slide viewers are of great importance. The use and existence of this resource needs to be promoted by the different actors (program directors, experts, residents and teachers) in pathology. An adequate integration into the academic curriculum is also a key factor to increase its efficacy.

We hope that a learning platform using WSI will also provide trainees with experience and confidence in the use and evaluation of this technology. It has been showed that diagnostic accuracy does not differ between the use of glass slides and the use of virtual microscopy [12,13]. However, the reluctance of users to embrace new technology has been shown to be one of the main factors limiting their implementation [14]. We hope that introducing this technology early during residency training will promote its future acceptance.

A collateral benefit of the project was the establishment of international partnerships

between French-speaking universities and pathologists with the common goal of promoting pathology education through the use of multi-media technology including whole slide imaging.

## List of abbreviations used

WSI: Whole-slide image; CFQCU: Conseil franco-québécois de coopération universitaire

## Competing interests

The authors declare having no competing interests in this study.

E.P. developed the contents and build the module with the financial support obtained from CFQCU http://www.cfqcu.org/.

## Authors' contributions

Emilie Perron, Maxime Battistella and Béatrice Vergier developed the contents, built the module and wrote the manuscript.

Maryse Fiche coordinates the Switzerland part of the network.

Philippe Bertheau initiated the network and coordinates its French part.

Bernard Têtu coordinates the Québec part of the network and wrote the manuscript.
